# Constrictive Pericarditis After Minimally Invasive Triple Valve Replacement: Insights From a Fatal Outcome

**DOI:** 10.7759/cureus.112536

**Published:** 2026-07-12

**Authors:** Santiago Luna-Alcala, Magali Herrera-Gomar, Sergio A Domínguez-Salazar, Javier Sánchez-Zavala, Javier A. Madrazo-Shiordia, Nalleli Duran López, Luis A Tirado-García, Humberto Martínez-Hernández, Guillermo Fernández de la Reguera-Fernández Del Campo

**Affiliations:** 1 Internal Medicine Department, Medica Sur Clinic and Foundation, Mexico City, MEX; 2 Coronary Care Department, Medica Sur Clinic and Foundation, Mexico City, MEX; 3 Pathology Department, Medica Sur Clinic and Foundation, Mexico City, MEX; 4 Cardiothoracic Surgery Department, Medica Sur Clinic and Foundation, Mexico City, MEX

**Keywords:** acute heart failure, cardiac magnetic resonance, cardiac pacemaker, constrictive, echocardiography, pleural effusion, restrictive, right-sided catheterization, thoracotomy, valve replacement

## Abstract

Triple valve surgery is performed in only a small minority of valvular procedures and carries substantial perioperative risk and a demanding postoperative course. Pericardial complications after cardiac surgery are well recognized, but progression to constrictive physiology following a minimally invasive triple valve operation is exceptionally uncommon and poorly characterized. We describe an elderly woman with longstanding hypertension, dyslipidemia, and paroxysmal atrial fibrillation who underwent minimally invasive replacement of the aortic, mitral, and tricuspid valves for combined multivalvular disease. Her recovery was complicated by recurrent pleural and pericardial effusions that evolved into constrictive pericarditis, with clinical and imaging features supporting the diagnosis, although distinguishing it from a restrictive process remained challenging despite complementary multimodality imaging and invasive hemodynamic assessment. Although pericardiectomy was undertaken, she deteriorated into refractory cardiogenic shock and died shortly afterwards. This report illustrates the difficulty of distinguishing constriction from restriction in the postoperative setting and underscores the value of close multidisciplinary follow-up after extensive valve surgery in older patients.

## Introduction

Multiple valvular heart disease (MVHD) affects up to one-fifth of patients with native valve disease and includes stenotic or regurgitant lesions in two or more valves or mixed lesions within a single valve [1]. Among surgical cases, double valve procedures -usually involving the aortic and mitral valves - account for approximately 11%, whereas triple valve replacement accounts for approximately 1% [1]. Constrictive pericarditis (CP) is an uncommon complication of cardiac surgery, arising from an exaggerated pericardial inflammatory response that progresses to fibrosis and impaired pericardial compliance; it often presents with progressive right-sided heart failure despite preserved biventricular systolic function and may closely resemble restrictive cardiomyopathy (RCM), prosthetic valve dysfunction, or other causes of postoperative heart failure, making diagnosis particularly challenging. Its differential diagnosis therefore includes RCM and postpericardiotomy syndrome (PPS), which share clinical features but differ in pathophysiology and imaging findings. In CP, a thick, non-compliant pericardium restricts diastolic filling despite preserved myocardial function, whereas RCM results from myocardial or endocardial stiffness that impairs ventricular relaxation [2]. CP is characterized by pericardial thickening, septal bounce, respiratory variation in ventricular filling, and discordant ventricular pressures, whereas RCM typically presents with a normal pericardium, impaired relaxation, minimal respiratory variation, and concordant pressures [2]. PPS is a postoperative inflammatory syndrome characterized by new pericardial effusion occurring one to two weeks after cardiac surgery, more commonly after aortic procedures. On CMR, CP demonstrates pericardial thickening and fibrosis, whereas PPS is characterized by pericardial inflammation and effusion without constrictive physiology [2,3]. In developed countries, CP most commonly follows idiopathic or viral pericarditis, with prior cardiac surgery and mediastinal radiation representing the next most frequent causes [2]. As a postoperative complication, CP is uncommon, occurring in approximately 0.2-0.3% of cases [4]. Its incidence after minimally invasive cardiac surgery remains unknown, and cases following minimally invasive triple-valve replacement have been reported only rarely, with the diagnostic course remaining poorly characterized. We present this case to illustrate its clinical presentation, multimodality imaging, and invasive hemodynamic findings, management, and outcome while highlighting the importance of maintaining a high index of suspicion and serial multimodality evaluation in patients with persistent postoperative right-sided heart failure.

## Case presentation

History of presentation

A 75-year-old woman was admitted to intermediate care after a year of worsening functional status, with exertional dyspnea (New York Heart Association, NYHA III), a decline in metabolic equivalents (METs) from 9 to 6.2, and nonspecific malaise. She reported an upper respiratory infection one month earlier, after which her symptoms worsened. She denied syncope, chest pain, edema, or orthopnea. On admission, she was afebrile (97.88°F), with an irregular pulse of 70 bpm, blood pressure of 126/63 mmHg, respiratory rate of 20 breaths/min, and oxygen saturation of 96% on room air. Cardiac examination revealed a late-peaking IV/VI systolic murmur at the right upper sternal border, obliterating S2 and radiating to the carotids, along with a high-pitched grade III/VI holosystolic murmur at the tricuspid area (inspiration) and mitral area (expiration).

Past medical history

Medical history included prediabetes, hypertension, dyslipidemia, and paroxysmal atrial fibrillation (AF; CHA₂DS₂-VASc 3). Chronic medications were isosorbide mononitrate, olmesartan, amiodarone, rosuvastatin, dapagliflozin, enoxaparin, and digoxin; amiodarone and digoxin provided rate and rhythm control, and enoxaparin was used as perioperative anticoagulation bridging. She had no prior cardiac interventions and no history of rheumatic fever; the valvular disease was degenerative. Family history was remarkable for heart failure and chronic kidney disease in her father.

Investigations

**Figure 1:**
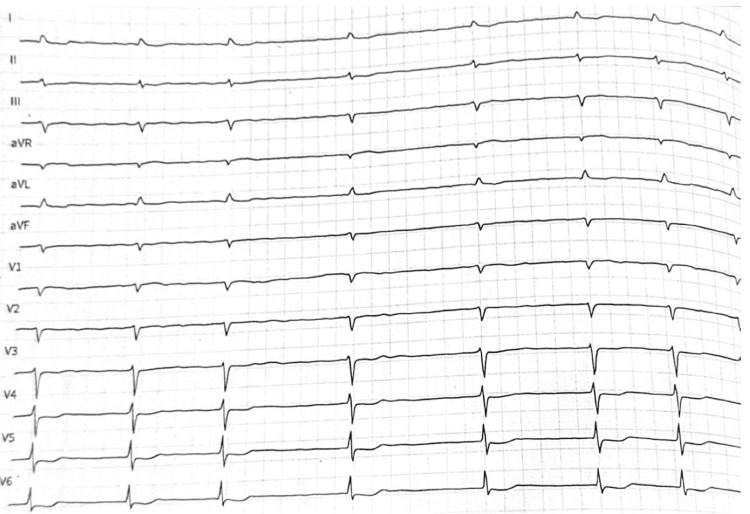
Electrocardiogram demonstrating atrial fibrillation. 12-lead electrocardiogram shows an irregular rhythm without distinct P waves, consistent with atrial fibrillation. QRS complexes appear low in amplitude across multiple leads.

Initial laboratory workup revealed microcytic hypochromic anemia, lymphopenia, and elevated NT-proBNP levels, with a negative anti-streptolysin O test (Table 1). The anemia was attributed to iron deficiency (low serum iron and transferrin saturation of 15.4%). It was mild and managed with preoperative optimization and perioperative transfusion support, without altering the surgical strategy. A 12-lead ECG demonstrated atrial fibrillation and left axis deviation (-54°) (Figure 1).

**Table 1:** Chronological timeline of the postoperative course. Ao: aorta/aortic pressure; AR: aortic regurgitation; AS: aortic stenosis; AV: aortic valve; bid: twice daily; bpm: beats per minute; CCU: coronary care unit; CPB: cardiopulmonary bypass; CPR: cardiopulmonary resuscitation; CRP: C-reactive protein; CT: computed tomography; ECG: electrocardiogram; LA: left atrium; LAAO: left atrial appendage occlusion; LV: left ventricle; LVEF: left ventricular ejection fraction; MR: mitral regurgitation; MV: mitral valve; NT-proBNP: N-terminal pro-B-type natriuretic peptide; PASP: pulmonary artery systolic pressure; RA: right atrium; ROSC: return of spontaneous circulation; RV: right ventricle; TOE: transoesophageal echocardiography; TR: tricuspid regurgitation; TTE: transthoracic echocardiography; TV: tricuspid valve; VExUS: venous excess ultrasound.

Date	Event/Intervention	Serial imaging, laboratory & haemodynamic findings	Status/outcome
PHASE 1 - Index admission and triple valve surgery (July 2025)			
16 Jul 2025	Admission; diagnostic coronary angiography	No significant coronary stenosis; baseline TTE: LVEF 65%, severe AR + moderate AS, severe MR and TR; both atria enlarged; NT-proBNP 3,488	Cleared for surgery
17 Jul 2025	Minimally invasive triple valve replacement via right minithoracotomy (AV Inspiris Resilia 21 mm + Nicks; MV Mitris 27 mm + LAAO; TV Mitris 27 mm)	CPB 308 min, cross-clamp 192 min; delta lactate 0.3, peak lactate 2; postoperative TOE: normofunctioning prostheses, no paravalvular leak	Admitted to CCU with vasopressor/inotrope support
18 Jul 2025	Pacemaker capture failure with hypotension → temporary transvenous pacemaker	12-lead ECG: pacing spikes without capture; high mediastinal drainage	Pacemaker-dependent; antibiotics switched to vancomycin
21 Jul 2025	Permanent VVI pacemaker (Biotronik)	Pacemaker-dependent at 50 bpm; TTE: paradoxical septal motion (early postoperative); prostheses normofunctioning	-
25 Jul 2025	Escalating inflammatory response	Leukocytosis, CRP 73.2, procalcitonin 0.39; chest CT: bilateral pleural effusion (right-predominant)	Empiric cefepime + vancomycin
26 Jul 2025	Right thoracocentesis (650 mL)	Transudate by Light's criteria	-
29-30 Jul 2025	Progressive congestion; diuretics + colchicine 0.5 mg bid started	TTE: anterior pericardial effusion (max 10 mm), hepatic-vein systolic-flow reversal, VExUS grade 3; CT: bilateral effusions, paracolic/pelvic fluid, abdominal-wall and vulvar oedema	First objective right-sided congestion
3 Aug 2025	Bilateral pleural drainage (R 800 mL, L 450 mL) + transthoracic catheter	Citrine fluid	-
7 Aug 2025	Discharge	-	-
PHASE 2 - Recurrence, diagnosis of constriction and pericardiectomy (September 2025)			
8 Sep 2025	Outpatient surveillance chest CT	Recurrent right pleural effusion (90%), left (10%)	Readmission planned
10 Sep 2025	Readmission; pleural drainage + pleurodesis (2,000 mL serous)	NT-proBNP 5,172; Kussmaul's sign present	-
15 Sep 2025	Cardiac magnetic resonance	Pericardial thickening adjacent to the RV free wall with septal bounce	Effusive → constrictive transition
17 Sep 2025	Right/left heart catheterization	Dip-and-plateau (“square-root”) sign; equalisation of end-diastolic pressures (LV, RV, RA); discordant respiratory variation (LV vs Ao); PASP 32 mmHg	Constrictive physiology confirmed
18 Sep 2025	Diagnosis of constrictive pericarditis confirmed; central venous access	—	Listed for pericardiectomy
19 Sep 2025	Anterolateral pericardiectomy	Thickened pericardium firmly adherent to epicardium; histology: fibrous tissue with lymphoplasmacytic infiltrate, reactive capillary proliferation, focal fibrin; no calcification/granulomas/malignancy	-
PHASE 3 - Refractory postoperative shock (September 2025)			
20 Sep 2025	Cardiogenic shock	Central venous saturation 32%, lactate 3.0; multiple vasopressors/inotropes started	-
23 Sep 2025	Refractory right-sided cardiogenic shock	Continuous renal replacement therapy initiated; NT-proBNP peak 24,366	-
24 Sep 2025	Ventricular fibrillation → CPR → Death	Refractory cardiogenic shock; asystole	Fatal outcome

**Figure 2:**
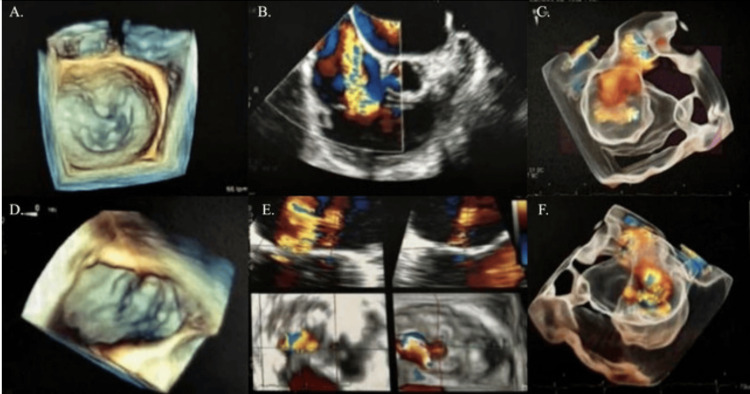
Multiplanar and 3D transoesophageal echocardiography of the mitral valve. A-D: 3D TOE en face reconstructions of the mitral valve, depicting leaflet morphology and coaptation from the atrial perspective. B: A 2D TOE view with color Doppler demonstrating abnormal transvalvular flow consistent with significant regurgitation. C and F: 3D color-rendered images illustrating the regurgitant jet origin and its spatial relationship to the mitral leaflets and annulus. E: Multiplanar reconstruction slices, allowing precise assessment of leaflet motion, jet direction, and mechanism of mitral regurgitation.

Transthoracic echocardiography (TTE) demonstrated preserved left ventricular (LV) ejection fraction (LVEF 65%), concentric remodeling (LV mass index: 74 g/m^2^; RWT: 0.43), and elevated LV filling pressures (septal E/e' 17.6, lateral E/e' 14.7). Right ventricular (RV) systolic function was normal (tricuspid annular plane systolic excursion (TAPSE): 23.6 mm, S' 14.5 cm/s, and right ventricular fractional area change (RVFAC): 41%). Both atria were enlarged (RA: 49.2 mL/m^2^; LA: 57.6 mL/m^2^), consistent with elevated left-sided filling pressures with preserved biventricular systolic function. A double aortic lesion - severe aortic regurgitation (AR) and moderate stenosis (AS) - was present, along with severe mitral and tricuspid regurgitation. Transesophageal echocardiography (TOE) confirmed thickened mitral leaflets (A2-A3/P2-P3) with three regurgitant jets: a small posterolateral jet (3D VCA 0.1 cm^2^), a larger central jet (3D VCA 0.56 cm^2^), and a smaller anteroseptal jet (Figures 2A-2F).

The tricuspid valve (TV) had thickened leaflets with a severe regurgitant jet (3D VCA: 0.49 cm^2^). The aortic valve (AV) showed thickened, fused cusps causing severe regurgitation (VCA: 0.45 cm^2^, RgV: 65 mL, EROA: 0.40 cm^2^, PHT: 180 ms) and moderate stenosis (AVA: 1.1 cm^2^, MPG: 20 mmHg, Vmax: 3.17 m/s) (Videos 1A-1F).

**Video 1:** Preoperative transoesophageal echocardiography. Multiplane preoperative transoesophageal echocardiography demonstrating the function of the aortic, mitral, and tricuspid valves. A: Mid-oesophageal four-chamber view at 0° showing thickened mitral leaflets with restricted motion. B: Mid-oesophageal mitral valve view with color Doppler at 120° demonstrating three mitral regurgitation (MR) jets: a small posterolateral jet, a large central jet, and a smaller anteroseptal jet. C: Mid-oesophageal tricuspid valve view with color Doppler at 135° showing markedly thickened tricuspid valve leaflets and a severe regurgitation jet. D: 3D atrial (en face from the left atrium) mitral valve view highlighting thickened mitral scallops (A2-A3/P2-P3) and the complex multijet regurgitation pattern. E: 3D ventricular (en face from the left ventricle) view showing the ventricular side of the thickened leaflets and the convergent MR jets. F: 3D en face view of the aortic valve demonstrating thickened and fused cusps.

**Figure 3:**
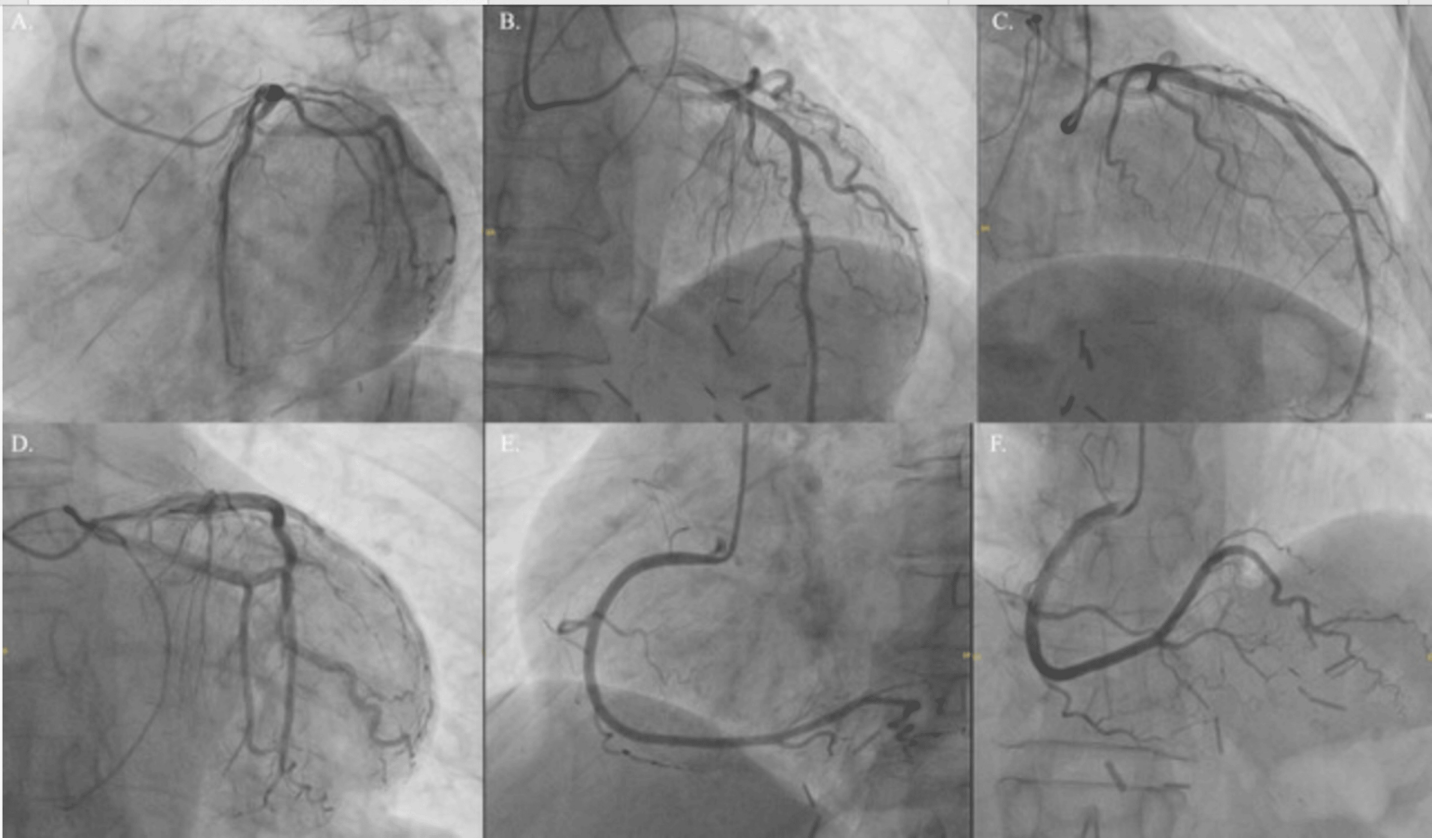
Visualization of coronary angiography. A-D: Left coronary system angiographic projections demonstrating unobstructed flow through the left main coronary artery, left anterior descending artery, and circumflex artery, with well-opacified distal branches. E-F: Right coronary artery angiograms revealing a dominant vessel with a normal course and preserved perfusion through its proximal, mid, and distal segments. Overall, the angiographic study depicts normal coronary anatomy without evidence of significant stenosis.

Coronary angiography revealed no significant stenosis (Figures 3A-3F, Videos 2A-2D).

**Video 2:** Coronary angiography. The study shows normal left and right coronary artery anatomy with unobstructed flow and no evidence of significant stenosis.

Management

The patient underwent minimally invasive triple valve surgery via right minithoracotomy, including AV replacement (Inspiris Resilia 21 mm) with Nicks annular enlargement, mitral valve (MV) replacement (Mitris Resilia 27 mm) with left atrial appendage occlusion (LAAO), and TV replacement (Mitris Resilia 27 mm) (Appendix, Supplement Videos 7-9). A minimally invasive approach was selected by the heart team to reduce surgical trauma in an elderly patient, avoid sternotomy-related wound complications, and facilitate recovery, while providing adequate exposure for all three valves. Bioprostheses were chosen on the basis of age, comorbidities, and bleeding risk, avoiding the lifelong anticoagulation burden of mechanical valves; the Nicks posterior enlargement prevented prosthesis-patient mismatch in the setting of a small aortic annulus (<21 mm). Cardiopulmonary bypass time was 308 minutes, with aortic cross-clamp time of 192 minutes, total diuresis of 1,250 mL, first-hour postoperative diuresis of 450 mL, and delta lactate of 0.3; the lactate trend remained stable throughout the perioperative period, indicating adequate tissue perfusion without evidence of a low-output state. Postoperative TOE showed a well-functioning aortic and mitral bioprosthesis without paravalvular leaks (AV: Vmax 2.2 m/s, MPG: 9 mmHg, EOA: 1.5 cm^2^) (MV: Vmax 1.5 m/s, MPG: 3 mmHg, PHT: 63 ms). The tricuspid biological prosthesis had mild regurgitation without paravalvular leak (MPG: 3 mmHg, TRV: 2.5 m/s, TRG: 25 mmHg) (Videos 3A-3B). She was admitted to the coronary care unit for monitoring and vasopressor support.

**Video 3:** Postoperative transoesophageal echocardiography of multivalve bioprostheses. Multiplane postoperative transoesophageal echocardiography demonstrating the function of the aortic, mitral, and tricuspid bioprostheses. A: Mid-oesophageal view at 0° showing the aortic and mitral bioprostheses. The aortic valve demonstrates normal function without paravalvular leak. The mitral bioprosthesis shows normal gradients and no paravalvular leak. B. Multiplane views at 135°, -21°, and 225° demonstrating the tricuspid bioprosthesis. The valve shows mild central regurgitation without paravalvular leak.

Outcome and follow-up

**Figure 4:**
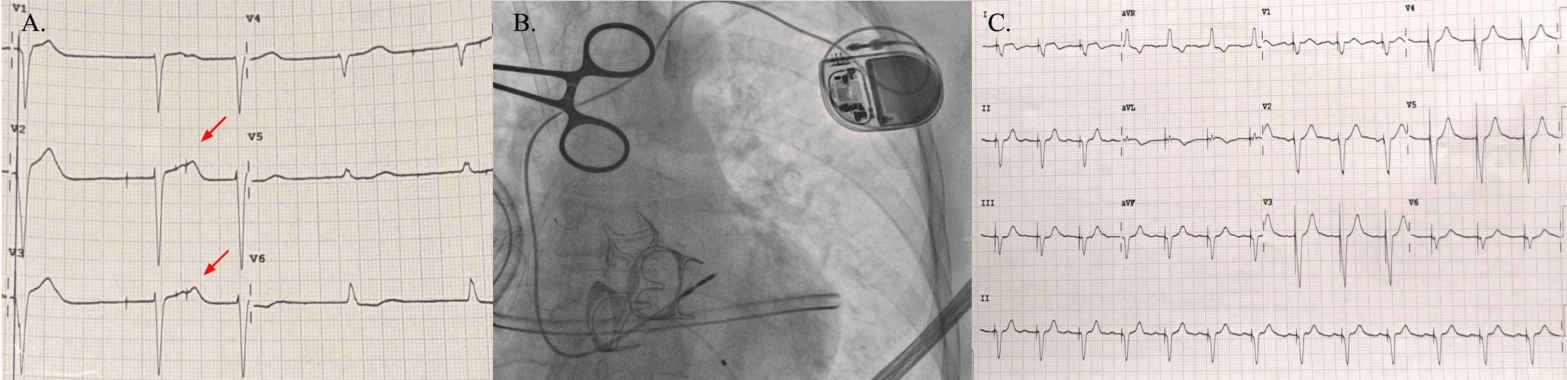
Electrocardiographic and fluoroscopic evaluation of pacemaker function. A: 12-lead electrocardiogram demonstrating failure of pacemaker capture, evidenced by pacing spikes (red arrows) not followed by ventricular depolarization. B: A fluoroscopic view of the pacemaker system, depicting appropriate lead positioning within the right heart chambers. C: Subsequent 12-lead electrocardiogram documenting restored pacemaker capture with consistent pacing spikes followed by corresponding ventricular complexes.

Hemodynamics showed CO of 3.5 L/min, SVR of 1,200 dyn·s/cm^5^, PCWP of 16 mmHg, and CVP of 3 mmHg. A dual-chamber, dual-sensed, dual-response (DDR) temporary pacemaker achieved full capture at 80 bpm, with dependency confirmed at 50 bpm; therefore, a permanent Biotronik VVI device was implanted (Figures 4A-4C).

**Figure 5:**
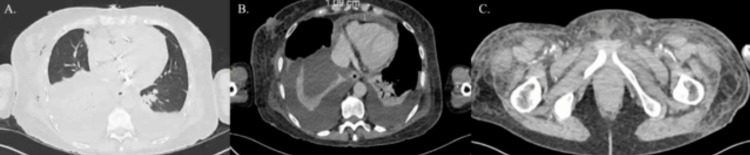
Thoracoabdominal computed tomography demonstrating pulmonary oedema and pericardial effusion. Axial computed tomography images. A: Diffuse bilateral pulmonary oedema characterized by smooth interlobular septal thickening and dependent alveolar opacities. B: Moderate pericardial effusion surrounding the heart. C: Pelvic soft-tissue oedema with prominent subcutaneous stranding, suggesting systemic inflammatory or edematous changes.

Persistent leukocytosis, with elevated C-reactive protein and procalcitonin, prompted seven days of empiric cefepime and vancomycin (Table 1). She developed genital lymphedema; thoracoabdominal computed tomography (CT) showed bilateral pleural effusion, 10 mm pericardial effusion, pulmonary artery dilation (2.5 cm), paracolic and pelvic fluid, and soft-tissue edema of the lower abdomen and vulva (Figures 5A-5C).

Thoracocentesis drained 800 mL (right) and 450 mL (left). Colchicine 0.5 mg b.i.d was initiated. Blood and urine cultures and rheumatologic studies, including complement levels, anti-dsDNA antibodies, and antinuclear antibodies, were negative (Table 1). She was discharged but re-admitted one month later with recurrent pleural effusion (90% right, 10% left), treated with drainage and pleurodesis, with a further rise in NT-proBNP. Kussmaul's sign was present (Video 4).

**Video 4:** Jugular venous pulse demonstrating Kussmaul's sign. With the onset of inspiration, instead of the expected fall in jugular venous pressure, there is a visible rise and distension of the jugular veins, which decrease again during expiration. This reflects impaired right ventricular filling and increased ventricular constraint.

**Figure 6:**
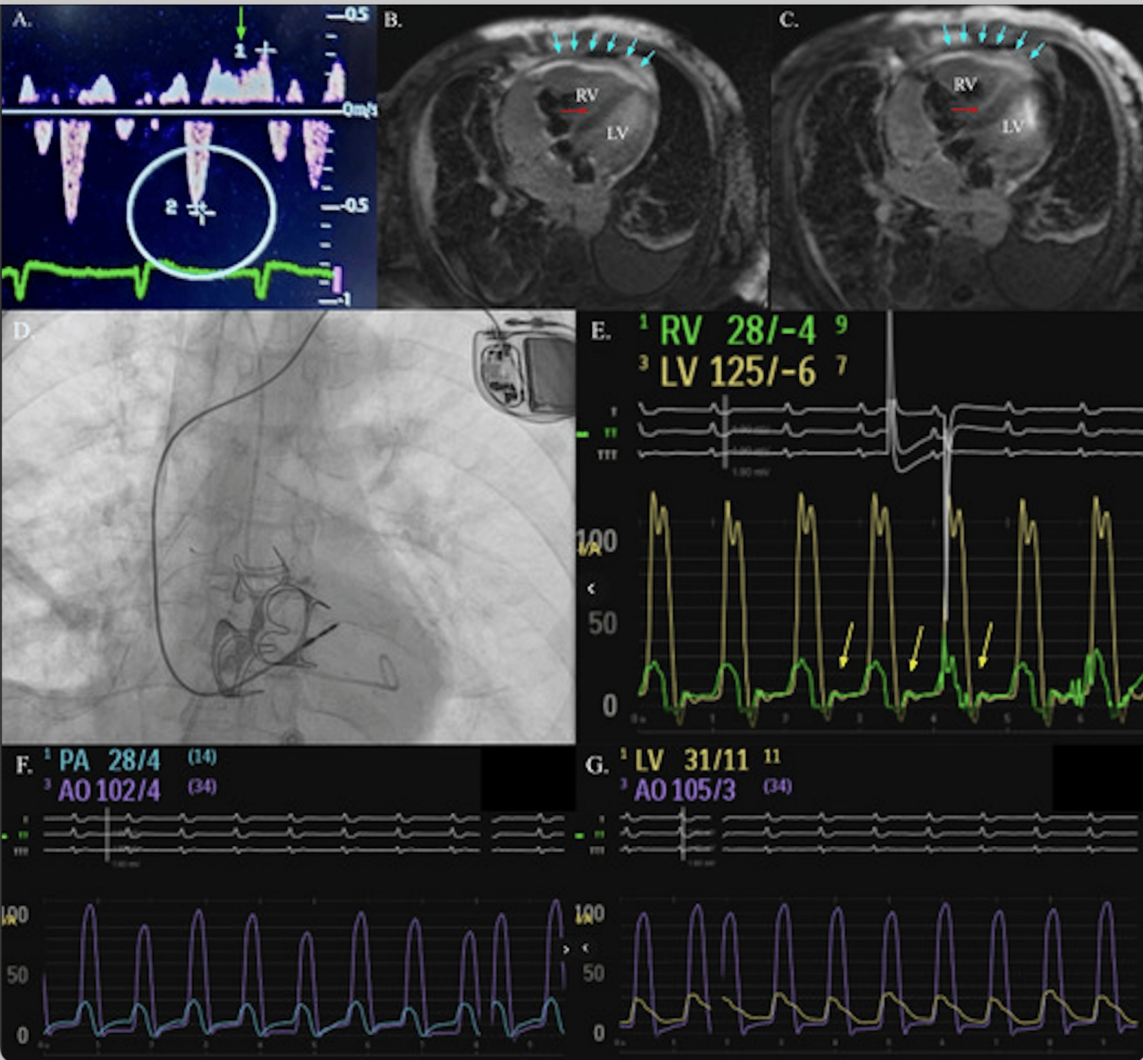
Multimodality assessment demonstrating constrictive physiology. Findings are grouped by their diagnostic weight for constrictive pericarditis. Supportive (non-invasive) findings. A: Doppler echocardiography showing prominent expiratory hepatic vein flow reversal (green arrow). B-C: Cardiac magnetic resonance imaging revealing pericardial thickening (blue arrows) with septal bounce (red arrow). Diagnostic (invasive) findings. D: Fluoroscopic view of catheter positioning during the haemodynamic study. E: Simultaneous right- and left-ventricular pressure tracings showing the dip-and-plateau ("square-root") sign in early diastole (yellow arrows). F-G: Simultaneous pressure tracings demonstrating marked respiratory discordance between left ventricular and aortic pressures, consistent with enhanced ventricular interdependence. The invasive tracings (E-G) were considered diagnostic of constriction, whereas the echocardiographic and CMR findings (A-C) were supportive.

Although the patient initially denied edema and orthopnea, objective signs of right-sided heart failure emerged postoperatively (including Kussmaul's sign, hepatic-vein systolic-flow reversal (VExUS grade 3), progressive lower-limb, abdominal-wall, and vulvar edema, recurrent effusions, and a rising NT-proBNP), indicating that right-sided congestion developed as a postoperative phenomenon rather than being present at baseline. These findings prompted echocardiographic evaluation for constriction: TTE showed septal bounce and hepatic-vein expiratory diastolic-flow reversal (ratio: 0.80), suggestive of constrictive pericarditis, although not all criteria were fulfilled (Figure 6A, Video 5).

**Video 5:** Transthoracic echocardiography showing septal bounce and a markedly thickened right ventricular free wall. A: Parasternal short-axis, mid-ventricular level. Circular left ventricle (LV) cavity in cross-section, with the interventricular septum visible centrally. The septum demonstrates abrupt, abnormal motion toward the LV during early diastole (septal bounce). The right ventricle (RV) free wall (anterior/lateral limb of the circular cross-section) appears markedly thickened and echogenic, compatible with persistent inflammatory change. B: Apical four-chamber view. The RV free wall is diffusely thickened with heterogeneous echogenicity. The interventricular septum shows paradoxical early-diastolic motion toward the LV consistent with septal bounce, with relative compression of the LV cavity in diastole.

CMR demonstrated septal bounce and pericardial thickening up to 5 mm with late gadolinium enhancement along the RV free wall (Figures 6B-6C, Video 6).

**Video 6:** Cardiac magnetic resonance demonstrating septal bounce, pericardial thickening, and right ventricular free-wall enhancement. Prominent septal bounce consistent with interventricular interdependence, together with pericardial thickening. Late gadolinium enhancement is present along the right ventricular (RV) free wall, indicating active pericardial and adjacent RV inflammatory involvement.

Right-heart catheterization confirmed a square-root sign and ventricular interdependence (Figures 6D-6G), in keeping with constrictive physiology; some indices, however, did not fully meet classic constrictive criteria, likely reflecting concomitant postoperative inflammation and altered loading conditions.

**Figure 7:**
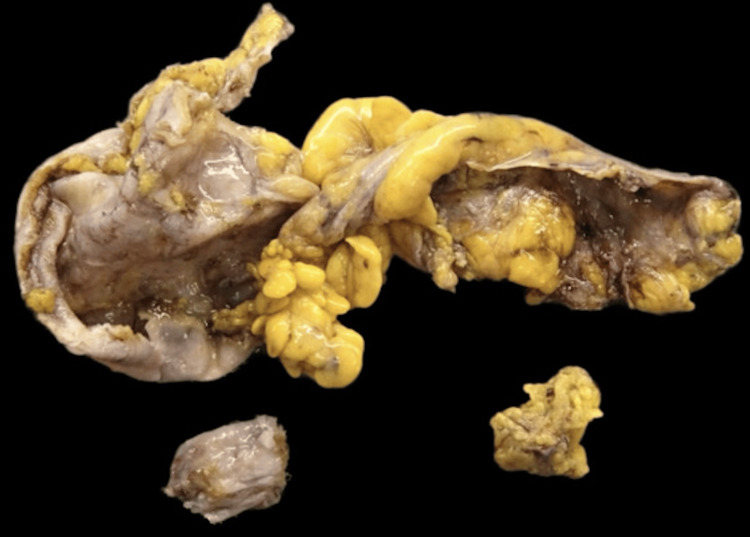
Macroscopic appearance of the anterolateral pericardiectomy specimen. An irregular laminar fragment measuring 13.5 × 8.0 cm. One surface appeared smooth and opaque, light brown in color with focal violaceous areas, while the opposite surface was rough and displayed a pink-brown hue with adherent adipose tissue. On sectioning, the tissue showed a fibrous consistency with a maximum thickness of approximately 0.2 cm.

An anterolateral pericardiectomy was performed (Appendix, Supplementary Videos 7-9). Histopathology showed a 13.5 x 8.0 x 0.2 cm pericardial sample with smooth external and rough internal surfaces, containing multilobulated yellow adipose tissue (Figure 7).

**Figure 8:**
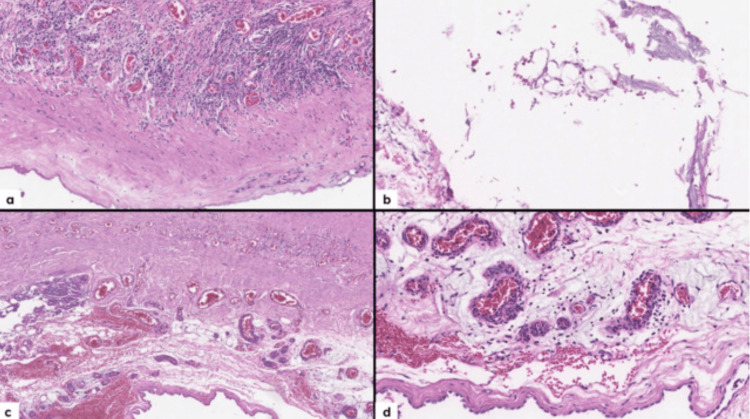
Photomicrograph, hematoxylin and eosin stain, ×40 magnification. A: Transmural pericardial fibrosis with a lymphocytic infiltrate and reactive capillary vascular proliferation. B: Focal fibrin deposits along the pericardial surface. C: Reactive capillary vascular proliferation with associated hemorrhage and adjacent loose connective tissue near the visceral pericardium. D: Loose connective tissue with congested vessels and prominent perivascular inflammation.

Microscopy revealed fibrous pericardial tissue with lymphoplasmocytic infiltrate and capillary proliferation (Figure 8), without calcification, granulomas, or malignancy; this pattern is consistent with an organizing, inflammatory-fibrosing pericarditis of postoperative origin, supporting a continuum from postpericardiotomy inflammation through effusive-constrictive to constrictive physiology.

Postoperatively, she developed cardiogenic shock requiring vasopressors, inotropes, and continuous renal replacement therapy, accompanied by a further rise in NT-proBNP and worsening thrombocytopenia (Table 1). She died five days later from refractory cardiogenic shock after ventricular fibrillation (Table 2).

**Table 2:** Laboratory investigations. Serial laboratory investigations across the clinical course. Columns correspond to presentation (preoperative baseline); the postoperative course after the triple valve surgery; the second admission on arrival; and the early period after the pericardiectomy. Values are shown against the local reference range; ↑ indicates a result above the reference range (increased) and ↓ a result below it (decreased); results within the reference range are shown without an arrow. In the post-triple valve surgery column, the haematological and inflammatory values correspond to the day of the peak inflammatory response, and the NT-proBNP is the peak postoperative value. An em dash (—) indicates a parameter not measured at that time point (C-reactive protein was not obtained preoperatively, and complement, anti-dsDNA, antinuclear, and anti-streptolysin O testing was performed only when clinically indicated). MCV: mean corpuscular volume; MCH: mean corpuscular haemoglobin; NT-proBNP: N-terminal pro-B-type natriuretic peptide; anti-dsDNA: anti-double-stranded DNA antibodies.

Parameter (Units)	Reference Range	Presentation (10 Jun 2025)	Post-triple valve surgery, peak (25 Jul 2025)	Readmission (10 Sep 2025)	Post-pericardiectomy (Sep 2025)
Hemoglobin (g/dL)	12.0-16.0	10.5 ↓	11.0 ↓	14.8	9.4 ↓
MCV (fL)	80-100	76.6 ↓	81.1	87	93.5
MCH (pg)	27-33	25.2 ↓	28.5	30.6	30.6
Platelets (×10³/µL)	150-450	417	153	163	92 ↓
Leukocytes (×10³/µL)	4.0-11.0	8.6	20.6 ↑	11.5 ↑	12.2 ↑
Lymphocytes (×10³/µL)	1.0-4.0	0.99 ↓	0.2 ↓	0.6 ↓	0.1 ↓
NT-proBNP (pg/mL)	< 125	3,488 ↑	8,518 ↑	5,172 ↑	24,366 ↑
C-reactive protein (mg/L)	< 5	—	73.2 ↑	66.4 ↑	44.0 ↑
Procalcitonin (ng/mL)	< 0.10	—	0.39 ↑	—	—
Complement C3 (mg/dL)	90-180	—	93.2	—	—
Complement C4 (mg/dL)	10-40	—	18.8	—	—
Anti-dsDNA (IU/mL)	< 30	—	10.9	—	—
Antinuclear antibodies (titer)	< 1:40	—	< 1:40	—	—
Anti-streptolysin O (IU/mL)	< 200	Negative	—	—	—

## Discussion

The management of MVHD requires an integrated assessment of lesion severity, valve interaction, ventricular response, and surgical feasibility, rather than evaluation of each valve lesion in isolation. Echocardiography remains the primary diagnostic tool [1,5]. In this case, TTE defined lesion mechanism, severity, and hemodynamic impact. Mixed aortic disease with moderate AS and severe AR warranted surgery in symptomatic patients regardless of LV function [4,6]. To avoid prosthesis-patient mismatch due to a small annulus (<21 mm), Nicks procedure was performed, as it provides safe posterior enlargement while avoiding the fibrous trigone and left atrium [7]. AV replacement was indicated for moderate AS due to planned MV and TV surgery, and aortic root replacement was unnecessary given the normal diameter (24 mm) [6,8]. Severe secondary mitral regurgitation was attributed to atrial dilation with preserved LVEF. MV surgery with LAAO was performed for persistent symptoms despite optimal medical therapy, and anticoagulation was continued for three months [5]. TV surgery was indicated for severe TR during left-sided procedures [5,6]. Valve replacement rather than repair was chosen for the mitral and tricuspid valves: the leaflets were markedly thickened and degenerative with a complex multijet regurgitation mechanism unfavorable for a durable repair, and a tricuspid annuloplasty ring was attempted intraoperatively but proved unsatisfactory, prompting conversion to replacement to secure a single definitive correction. A pacemaker was required for conduction disturbances, reported in up to 14% of cases, such as atrioventricular block [6]. Bioprostheses were selected based on age, comorbidities, and bleeding risk [5,6]. Transcatheter aortic valve implantation (TAVI) was not favored because it carries a higher risk of vascular complications, paravalvular regurgitation, and reintervention [5].

CP after triple-valve surgery is rare, and no incidence data exist for minimally invasive approaches [4]. After cardiac surgery, new pericardial effusion and inflammation are common and most often reflect self-limited PPS, which is managed conservatively with anti-inflammatory therapy. The clinical challenge is to identify the minority of patients in whom the process progresses to constriction, since refractory constrictive (or effusive-constrictive) pericarditis is relieved only by pericardiectomy, whereas RCM, despite similar hemodynamics, derives no benefit from pericardial surgery and is managed medically. In the postoperative setting, this distinction determines whether to continue anti-inflammatory therapy, to withhold surgery, or to proceed to pericardiectomy; misclassification risks either an unnecessarily high-risk operation or delayed definitive treatment. Constriction results from a thickened, fibrotic, and often calcified pericardium that limits diastolic filling, whereas CP refers to constriction accompanied by active pericardial inflammation [9]. It typically presents as right-sided heart failure with preserved biventricular systolic function. Echocardiography is the cornerstone of diagnosis. Septal bounce and a hepatic vein expiratory diastolic reversal ratio >0.78 provide 87% sensitivity and 91% specificity. Other findings include a medial e′ velocity >8 cm/s and respiratory variation in mitral (>25%) and tricuspid (>40%) inflow velocities; when all are present, specificity increases to 97%, although sensitivity decreases to 64% [9].

Despite multimodality imaging, overlapping features between CP and RCM persisted, particularly in the context of postoperative inflammation and effusion; invasive hemodynamics were therefore decisive in favoring a constrictive physiology (Table 3). Cardiac catheterization established the diagnosis by demonstrating a “square-root” sign, reflecting rapid early diastolic filling that was abruptly limited by a non-compliant pericardium, producing an early pressure dip followed by a mid-to-late diastolic plateau [10]. Effusive-constrictive forms may resolve spontaneously or respond to anti-inflammatory therapy, but refractory cases require visceral pericardiectomy because the visceral pericardium is the principal site of constriction [10]. In this patient, recurrent effusions developed approximately two weeks after minimally invasive triple-valve surgery, whereas constrictive physiology was confirmed invasively at nine weeks postoperatively, immediately preceding pericardiectomy. Surgery was undertaken because right-sided heart failure progressed despite anti-inflammatory therapy and diuresis, although pericardiectomy carries a contemporary perioperative mortality of 6-10% [9].

Alternative causes of postoperative right-sided heart failure were systematically excluded. Prosthetic dysfunction was ruled out by serial TTE and TOE, demonstrating normally functioning prosthetic valves without paravalvular leak. Pulmonary hypertension was excluded based on an invasive systolic pulmonary artery pressure of 32 mmHg and a low postoperative echocardiographic probability. RCM was considered less likely given pericardial thickening, septal bounce, respiratory discordance, and a dip-and-plateau sign, although overlapping features remained. PPS was considered the initiating process rather than the final diagnosis because, although the early pericardial effusions and inflammatory markers were consistent with this entity, the clinical course failed to improve with anti-inflammatory therapy and instead progressed to established constrictive physiology. A postoperative low-output state was excluded by preserved biventricular systolic function, an adequate cardiac output (3.5 L/min), and a stable perioperative lactate trend, indicating that congestion resulted from impaired diastolic filling rather than pump failure. Infection was also considered because of postoperative leukocytosis and elevated C-reactive protein and procalcitonin levels; however, blood and urine cultures remained negative, and these abnormalities were attributed to postoperative pericardial inflammation and the surgical insult rather than sepsis. Overall, the convergence of multimodality imaging and invasive hemodynamic findings (including pericardial thickening with late gadolinium enhancement, septal bounce, hepatic vein expiratory diastolic flow reversal, and a square-root sign with ventricular interdependence) supported CP as the unifying diagnosis (Table 3).

**Table 3:** Findings supporting versus opposing or uncertain for constrictive physiology. CMR: cardiac magnetic resonance; LV: left ventricle; RV: right ventricle

Features supporting constriction	Features opposing or uncertain
Septal bounce on echocardiography and CMR. Hepatic-vein expiratory diastolic-flow reversal ratio 0.80 (>0.78). Kussmaul's sign. Pericardial thickening up to 5 mm with late gadolinium enhancement along the RV free wall (CMR). Dip-and-plateau (“square-root”) sign on invasive hemodynamics. Ventricular interdependence with respiratory discordance (LV vs aortic pressures). Preserved biventricular systolic function. Histology: organizing fibrous pericarditis with lymphoplasmacytic infiltrate and reactive capillary proliferation.	Persistent overlap with restrictive physiology in the postoperative inflammatory setting. Some invasive indices did not fully meet classic constrictive criteria (incomplete pressure equalization; loading-dependent tracings). Active inflammation and recurrent effusions (effusive-constrictive rather than chronic calcific constriction). Absence of pericardial calcification. Atrial fibrillation and vasoactive/ventilatory support confounding beat-to-beat respiratory analysis. Elevated left-ventricular filling pressures (E/e′) that could also reflect a myocardial component.

Mechanistically, we propose a continuum in which pericardiotomy-related inflammation produced recurrent pericardial effusions and organizing fibrinous pericarditis that progressed to effusive-constrictive and ultimately constrictive physiology. Pericardiectomy was pursued despite the incomplete fulfilment of all non-invasive diagnostic criteria because invasive hemodynamic assessment demonstrated equalization of end-diastolic pressures, ventricular interdependence, and a square-root sign. The fatal outcome was likely multifactorial, reflecting advanced age, prolonged cardiopulmonary bypass and aortic cross-clamp times (308 and 192 minutes, respectively), repeated inflammatory insults, pacemaker dependence, and delayed diagnostic certainty. Whether earlier anti-inflammatory therapy or earlier pericardiectomy might have altered the clinical course remains speculative. As a single-patient report, this case cannot establish causality and has limited generalizability.

## Conclusions

This case underscores the need for guideline-directed evaluation in MVHD, with echocardiography serving as the primary tool for assessing lesion severity and determining surgical timing. It highlights the challenge of distinguishing CP from RCM and the value of multimodality imaging. Postoperative complications reflect the high-risk nature of extensive valve surgery in elderly patients, reinforcing the importance of individualized, multidisciplinary management. Although CP followed minimally invasive triple valve surgery in this patient, a single case cannot establish a causal relationship, particularly as no incidence data exist for minimally invasive approaches. In patients with persistent postoperative pleural or pericardial effusions, or unexplained right-sided heart failure after cardiac surgery, we suggest a low threshold for serial multimodality assessment (including repeated echocardiography, early cardiac magnetic resonance imaging, and, when non-invasive findings are inconclusive, invasive hemodynamic evaluation) coordinated through a multidisciplinary team. These observations derive from an individual report and should not be generalized; dedicated studies are needed to define the incidence, risk factors, and optimal management of CP after minimally invasive multivalve surgery.

## References

[1] Unger P, Pibarot P, Tribouilloy C, Lancellotti P, Maisano F, Iung B, Piérard L (2018). Multiple and mixed valvular heart diseases: pathophysiology, imaging, and management. Circ Cardiovasc Imaging.

[2] Garcia MJ (2016). Constrictive pericarditis versus restrictive cardiomyopathy?. J Am Coll Cardiol.

[3] Gabaldo K, Sutlić Ž, Mišković D (2019). Postpericardiotomy syndrome incidence, diagnostic and treatment strategies: experience at two collaborative centers. Acta Clin Croat.

[4] Cimino JJ, Kogan AD (1989). Constrictive pericarditis after cardiac surgery: report of three cases and review of the literature. Am Heart J.

[5] Otto CM, Nishimura RA, Bonow RO (2021). 2020 ACC/AHA guideline for the management of patients with valvular heart disease: a report of the American College of Cardiology/American Heart Association Joint Committee on Clinical Practice Guidelines. Circulation.

[6] Praz F, Borger MA, Lanz J (2025). 2025 ESC/EACTS guidelines for the management of valvular heart disease: developed by the task force for the management of valvular heart disease of the European Society of Cardiology (ESC) and the European Association for Cardio-Thoracic Surgery (EACTS). Eur Heart J.

[7] Massias SA, Pittams A, Mohamed M, Ahmed S, Younas H, Harky A (2021). Aortic root enlargement: when and how. J Card Surg.

[8] Mazzolai L, Teixido-Tura G, Lanzi S (2024). 2024 ESC guidelines for the management of peripheral arterial and aortic diseases: developed by the task force on the management of peripheral arterial and aortic diseases of the European Society of Cardiology (ESC). Endorsed by the European Association for Cardio-Thoracic Surgery (EACTS), the European Reference Network on Rare Multisystemic Vascular Diseases (VASCERN), and the European Society of Vascular Medicine (ESVM). Eur Heart J.

[9] Schulz-Menger J, Collini V, Gröschel J (2025). 2025 ESC guidelines for the management of myocarditis and pericarditis: developed by the task force for the management of myocarditis and pericarditis of the European Society of Cardiology (ESC). Endorsed by the Association for European Paediatric and Congenital Cardiology (AEPC) and the European Association for Cardio-Thoracic Surgery (EACTS). Eur Heart J.

[10] Doshi S, Ramakrishnan S, Gupta SK (2015). Invasive hemodynamics of constrictive pericarditis. Indian Heart J.

